# Neuroregenerative Treatment With Radio Electric Asymmetric Conveyer Technology in Advanced Childhood Cerebral X-linked Adrenoleukodystrophy: Clinical Stabilization and Electroencephalographic Evidence of Cortico-Subcortical Reorganization

**DOI:** 10.7759/cureus.95560

**Published:** 2025-10-28

**Authors:** Vania Fontani, Arianna Rinaldi, Salvatore Rinaldi

**Affiliations:** 1 Department of Research, Rinaldi Fontani Foundation, Florence, ITA; 2 Department of Regenerative Medicine, Rinaldi Fontani Foundation, Florence, ITA; 3 Department of Research, Rinaldi Fontani Institute, Florence, ITA; 4 Department of Adaptive Neuro Psycho Physio Pathology and Neuro Psycho Physical Optimization, Rinaldi Fontani Institute, Florence, ITA; 5 Department of Regenerative Medicine, Rinaldi Fontani Institute, Florence, ITA

**Keywords:** bioelectrical modulation, brain mapping, care guidelines, childhood cerebral ald, eeg, neuroplasticity, neuroprotection, neuroregeneration, reac, x-linked adrenoleukodystrophy

## Abstract

We describe the case of a male child with advanced childhood cerebral X-linked adrenoleukodystrophy (Loes score 15) who underwent five cycles of radio electric asymmetric conveyer (REAC) neuro regenerative (RGN-N) treatment over a two-year period. Clinical assessments and neurophysiological recordings were performed at baseline and during follow-up. The pre-treatment electroencephalogram (EEG) and brain mapping showed disorganized background activity for age, continuous bilateral frontal slowing, and abundant epileptiform discharges with right-sided predominance. Post-treatment evaluations performed four months after the last cycle revealed background activity appropriate for age, a reduction of slowing to intermittent focal changes confined to the left frontal region, and a restriction of epileptiform activity to a small area involving the right centro-rolandic region. Sustained clinical stabilization was observed, with decreased spasticity (Modified Ashworth Scale from 3 to 1), recovery of visual tracking, improved emotional responsiveness, and resolution of the conditions that had previously required repeated hospitalizations, along with discontinuation of oxygen therapy and airway suctioning. These findings contrast with the expected course of progressive deterioration in this condition and provide objective neurophysiological evidence of cortical reorganization associated with REAC RGN-N treatment. Further studies are warranted to explore the potential of this therapeutic approach in advanced stages of neurodegenerative disorders.

## Introduction

Childhood cerebral X-linked adrenoleukodystrophy (X-ALD) is a rare, inherited neurodegenerative disorder caused by pathogenic mutations in the ATP-binding cassette sub-family D member 1 (ABCD1) gene, which encodes the peroxisomal membrane transporter adrenoleukodystrophy protein (ALDP). This defect impairs peroxisomal β-oxidation of very-long-chain fatty acids (VLCFAs), leading to their accumulation in plasma and tissues, particularly in the central nervous system, adrenal cortex, and testes [1]. The childhood cerebral form is the most severe phenotype, typically manifesting between four and 10 years of age with behavioural changes, cognitive decline, motor impairment, visual and auditory deficits, and rapid progression to severe disability and death within a few years of symptom onset [2].

Neuroimaging classically reveals symmetric parieto-occipital white matter demyelination, with gadolinium enhancement indicating active neuroinflammation and blood-brain barrier disruption [3]. The natural history in advanced childhood cerebral X-ALD is characterized by relentless neurological deterioration, progressive worsening of neurophysiological parameters [4], and increasing Loes scores [3], a semi-quantitative MRI-based scale ranging from 0 (normal brain) to 34 (maximum involvement), which reflects the extent and severity of demyelinating lesions.

Hematopoietic stem cell transplantation (HSCT) is the only intervention currently capable of modifying the disease course, but its efficacy is restricted to early stages before significant neurological and radiological damage has occurred [5]. Once the disease reaches an advanced stage, HSCT and other available therapies are essentially palliative, aimed at symptom control rather than disease modification.

No non-invasive intervention has previously been shown to produce sustained clinical improvements together with objective neurophysiological changes in advanced childhood cerebral X-ALD. Electroencephalography (EEG) and quantitative brain mapping are particularly valuable in this context, as they provide objective measures of cortical organisation, functional connectivity, and epileptiform activity parameters that normally deteriorate steadily as the disease progresses. In X-ALD, increased epileptiform activity and diffuse slowing on EEG have been associated with functional decline, making improvement in these parameters not only measurable but clinically relevant [3].

Radio electric asymmetric conveyor (REAC) technology is a non-invasive neuromodulation and biomodulation platform designed to interact with the body's endogenous bioelectrical activity through asymmetrically conveyed radioelectric fields. By restoring altered bioelectrical gradients in target tissues, REAC protocols aim to promote reparative and regenerative processes [6-9]. Preclinical studies have demonstrated that REAC neuroregenerative protocols can modulate pathological neuroinflammation in models of Alzheimer's disease [10] and Parkinson's disease [11], downregulating pro-inflammatory cytokines and glial activation while upregulating anti-inflammatory mediators. At the cellular level, REAC exposure induces neuronal differentiation, increases expression of neurogenic transcription factors (neurogenin-1), structural proteins (β3-tubulin), and neurotrophic factors (NGF), supporting neuritogenesis and synaptic maturation [11]. Functional MRI studies in humans have shown that even a single REAC neuromodulation session can produce lasting changes in brain activation patterns, consistent with reorganisation and optimisation of functional networks [12].

Clinically, REAC neuroregenerative (RGN-N) protocols have shown potential benefits in various neurodegenerative [13,14] and neurodevelopmental conditions [15,16]. In the context of X-ALD, two previously published paediatric case reports have documented sustained improvements in motor, cognitive, and swallowing functions following cycles of REAC RGN-N, even in advanced stages where no other active therapeutic options were available [9,17]. However, these reports relied solely on clinical observation without objective neurophysiological confirmation.

The present case report describes a patient with advanced childhood cerebral X-ALD who underwent repeated REAC RGN-N treatment cycles over a two-year period. For the first time in this disease context, sustained clinical improvements were accompanied by pre- and post-treatment EEG and quantitative brain mapping evidence of cortico-subcortical functional reorganisation, contrasting sharply with the progressive deterioration typically observed in the advanced stage of the disease.

## Case presentation

The patient is a male child born in June 2012 after an uneventful full-term pregnancy, with normal early psychomotor development. There was no family history of X-linked adrenoleukodystrophy or other neurodegenerative or metabolic disorders, nor any consanguinity. Growth and developmental milestones were within the expected range during infancy and early childhood. School performance was initially age-appropriate.

In April 2022, at the age of nine years and 10 months, the child was referred for neurological evaluation due to progressive behavioural disturbances, episodes of irritability, and reduced attention span. Clinical examination was unremarkable except for subtle behavioural changes. Neuroimaging and laboratory studies were performed. Gadolinium enhancement confirmed active neuroinflammatory activity. The Loes score was calculated as 15, indicating advanced disease.

The diagnosis of X-linked adrenoleukodystrophy (X-ALD) had been previously established based on clinical findings, brain MRI

Following the first REAC RGN-N cycle in June 2023, there was a marked reduction in seizure frequency and severity. Persistent crying and distress ceased, sedation was no longer required, and the need for airway suctioning decreased substantially. Oxygen supplementation was reduced, and the patient remained more stable between episodes.

After the second cycle in October 2023, these improvements were maintained and further consolidated. The last hospitalisation occurred in January 2024 for a destabilising seizure. Following the third cycle in June 2024, no further hospital admissions were required. Clinical stability was accompanied by a progressive reduction in the need for continuous medical supervision at home.

By the time of the fourth cycle in October 2024, 24-hour home care was no longer necessary. Spasticity, systematically assessed using the Modified Ashworth Scale (MAS) [18], a clinical scale that quantifies muscle tone from 0 (no increase) to 4 (rigid limb in flexion or extension), had decreased from a score of 3 to 1, enabling more comfortable positioning in bed and allowing passive lower limb mobilisation during physiotherapy. Visual tracking of people and objects reappeared, and emotional responsiveness improved, with the patient expressing displeasure, contentment, or interest through facial expressions and vocalisations.

The fifth cycle in May 2025 consolidated these gains. At four months after the last cycle (September 2025), the patient remained free from hospitalisations, did not require oxygen therapy, and no longer needed airway suctioning or constant medical supervision. Multidisciplinary evaluations, corroborated by caregiver reports, confirmed sustained improvements in muscle tone, voluntary motor participation, visual engagement, and socio-emotional interaction.

**Figure 1:**
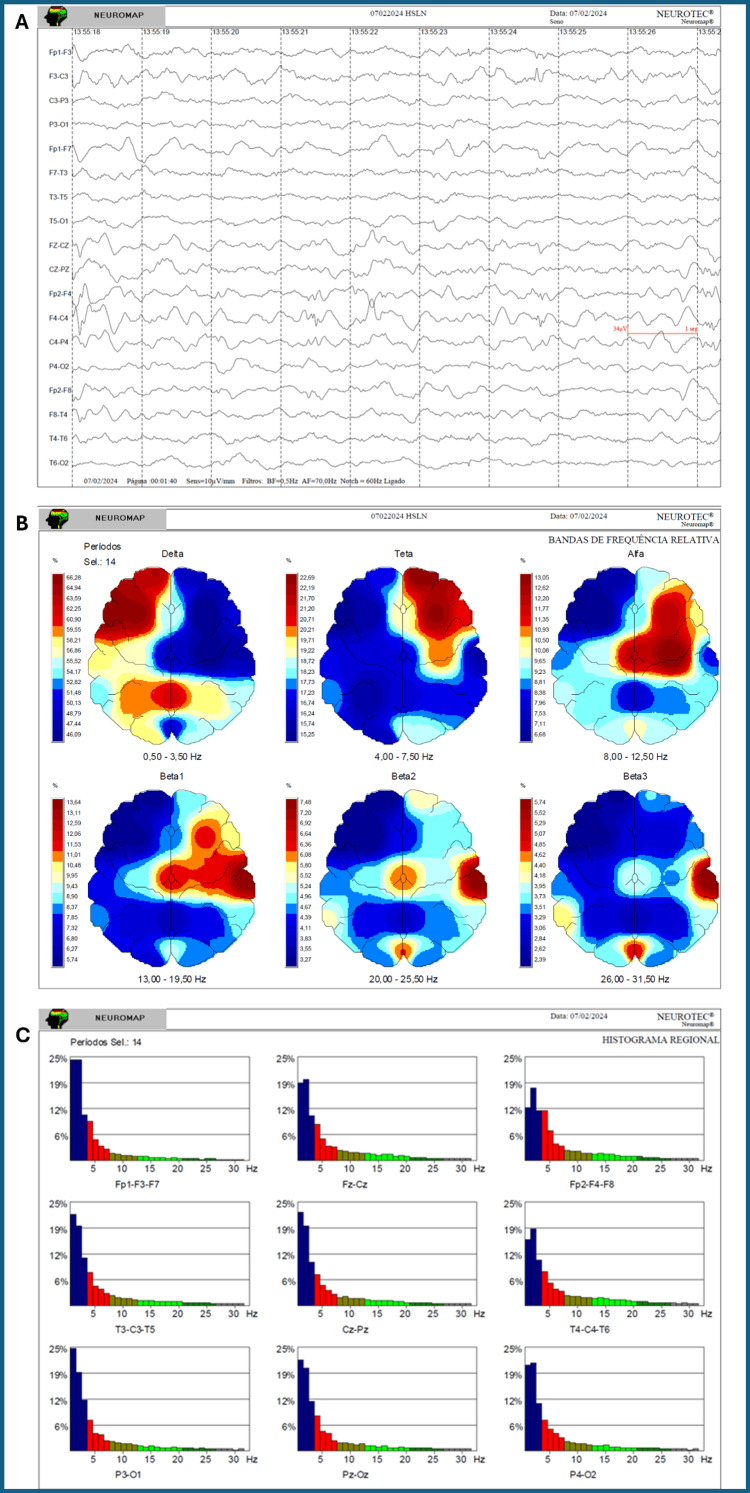
Baseline EEG and quantitative brain mapping (February 2024) (A) EEG recording showing background activity disorganised for age, continuous bilateral frontal slowing, and abundant epileptiform discharges with right frontal predominance; (B) Quantitative brain mapping demonstrates marked predominance of delta activity in the bilateral frontal regions, associated with reduced representation of faster rhythms; (C) Regional spectral histograms confirm the predominance of slow-wave activity, particularly in anterior regions.

Neurophysiological assessment with EEG and quantitative brain mapping, performed in February 2024 (Figure 1) before the most recent treatment cycle and in September 2025 afterwards, demonstrated substantial changes.

**Figure 2:**
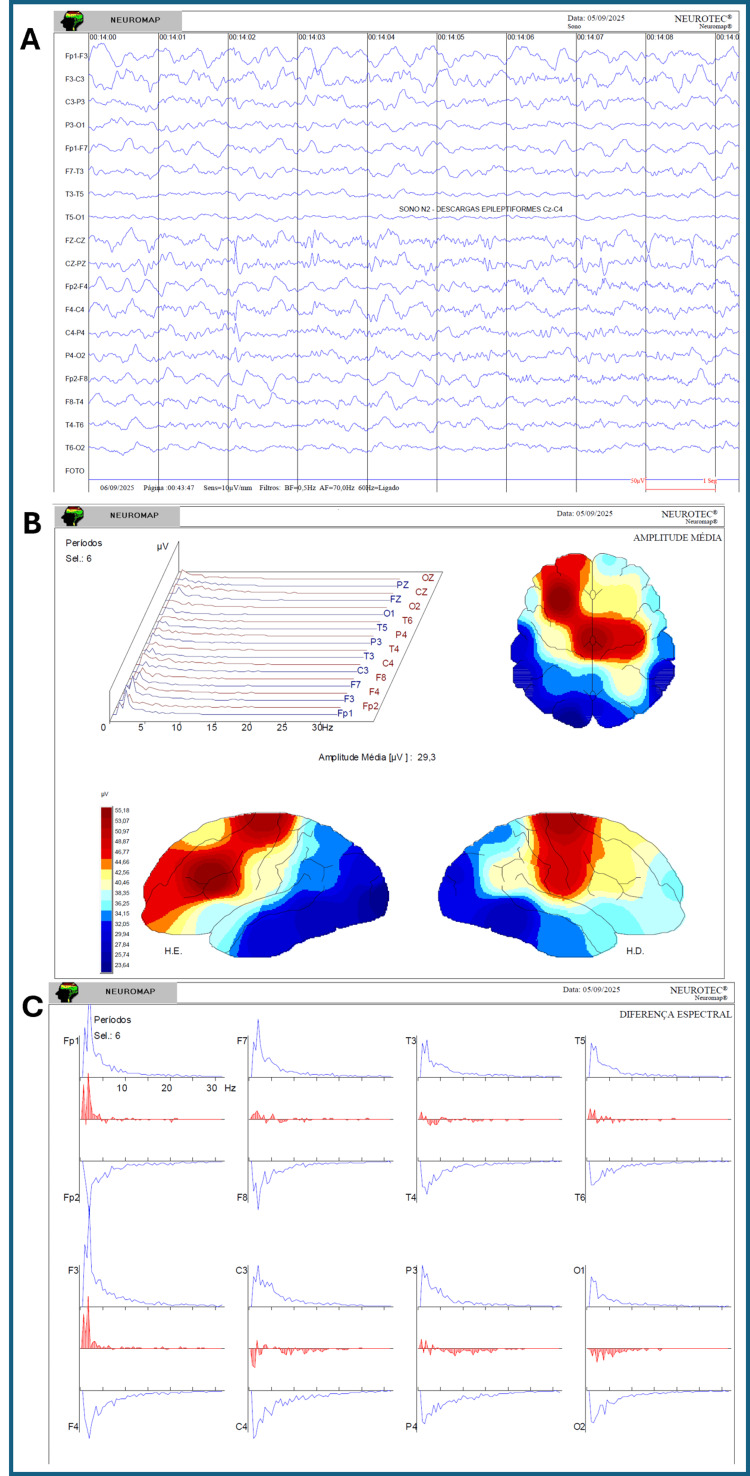
Post-treatment EEG and quantitative brain mapping (September 2025) (A) EEG recording showing background activity appropriate for age, slowing reduced to intermittent focal changes in the left frontal region, and epileptiform discharges restricted to a small, circumscribed area involving the right centro-rolandic region; (B) Quantitative brain mapping demonstrates reduced frontal delta predominance and a more balanced distribution of frequency bands, indicative of improved cortical organization; (C) Regional spectral histograms show a marked reduction in slow-wave predominance compared to baseline, with a relative increase in faster frequency components.

The post-treatment EEG was obtained four months after the fifth cycle, providing evidence of medium-term persistence of the neurophysiological effects. The pre-treatment EEG showed disorganised background activity for age, continuous bilateral frontal slowing, and abundant bilateral epileptiform discharges with right-sided predominance. Post-treatment recordings revealed background activity appropriate for age, slowing reduced to intermittent focal changes limited to the left frontal region, and epileptiform activity restricted to a smaller, more circumscribed area involving the central midline and right rolandic regions. No electrographic seizures were recorded in either study. These neurophysiological improvements corresponded closely to the observed clinical stabilisation and functional recovery, in clear contrast to the progressive deterioration expected in advanced X-ALD (Figure 2).

## Discussion

Childhood cerebral X-linked adrenoleukodystrophy (X-ALD) is one of the most aggressive leukodystrophies, with a natural course characterised by rapid neurological deterioration, progressive cerebral demyelination, and poor prognosis. In the advanced stage, patients typically experience continuous loss of function, further disorganisation of background EEG activity, increasing diffuse slowing, and a wider distribution of epileptiform abnormalities. Against this expected trajectory, the sustained clinical stabilisation and neurophysiological improvements observed in the present case are exceptional.

Two previously published paediatric case reports of advanced X-ALD treated with REAC RGN-N have documented durable clinical benefits, including reductions in spasticity, as assessed by the MAS [18], improvements in swallowing and motor performance, and enhanced cognitive and emotional responsiveness [9]. However, none of these reports included objective neurophysiological follow-up data, making the present case the first to provide EEG and quantitative brain mapping evidence of improved cortical organisation and reduced extent and distribution of epileptiform activity after REAC RGN-N treatment in advanced X-ALD.

The persistence of these changes over a two-year period suggests that REAC RGN-N may exert neuroprotective and neuroregenerative effects through mechanisms demonstrated in preclinical studies. In murine models of Alzheimer's disease and Parkinson's disease, REAC protocols have been shown to modulate pathological neuroinflammation, downregulating pro-inflammatory mediators such as interleukin-1 beta (IL-1β), interleukin-6 (IL-6), tumor necrosis factor-alpha (TNF-α), and inducible nitric oxide synthase (iNOS), while upregulating anti-inflammatory factors including interleukin-10 (IL-10) and suppressor of cytokine signaling-1 (SOCS-1) [19]. These effects were associated with reduced astroglial and microglial activation and preservation of neuronal integrity.

At the cellular level, REAC exposure promotes neuronal differentiation [20], neuritogenesis, and synaptic maturation, with increased expression of neurogenic transcription factors such as neurogenin-1, structural proteins such as β3-tubulin, and neurotrophic factors such as NGF [11]. In human studies, functional MRI analyses have shown that REAC neuromodulation can produce lasting changes in brain activation patterns, consistent with optimisation and reorganisation of functional networks [12].

This combination of mechanisms, reduction of neuroinflammation, promotion of neuronal plasticity, and functional network reorganization provides a biologically plausible explanation for the sustained clinical and EEG improvements observed in this patient, despite the underlying progressive genetic pathology.

Importantly, no adverse effects were reported across five treatment cycles in a medically fragile individual, supporting the long-term safety and tolerability of the protocol. While the findings from a single case cannot establish causality, the convergence of objective neurophysiological evidence, sustained clinical benefit, and mechanistic plausibility strengthens the rationale for further investigation of REAC RGN-N in advanced X-ALD. Given the uniformly poor prognosis of advanced childhood cerebral X-ALD, further research is urgently needed, ideally registry-based or coordinated multi-centre observational studies, to confirm these results. Exploratory evaluation in other leukodystrophies with similar inflammatory-demyelinating features could be considered.

## Conclusions

This case describes a child with advanced childhood cerebral X-ALD who experienced sustained clinical stabilisation and functional recovery following repeated cycles of REAC RGN-N neuroregenerative treatment. Improvements were documented through multidisciplinary clinical evaluations and caregiver reports and objectively confirmed by pre- and post-treatment EEG and quantitative brain mapping, which demonstrated enhanced cortical organisation and a reduction in both the extent and distribution of epileptiform activity.

This case provides the first objective neurophysiological evidence of sustained cortical reorganisation after REAC RGN-N treatment in advanced childhood cerebral X-ALD, highlighting its potential relevance in a condition with otherwise relentless progression.
